# Brain pericytes increase the lipopolysaccharide-enhanced transcytosis of HIV-1 free virus across the *in vitro* blood–brain barrier: evidence for cytokine-mediated pericyte-endothelial cell crosstalk

**DOI:** 10.1186/2045-8118-10-23

**Published:** 2013-07-01

**Authors:** Shinya Dohgu, William A Banks

**Affiliations:** 1 Department of Pharmaceutical Care and Health Sciences, Faculty of Pharmaceutical Sciences, Fukuoka University, Fukuoka, Japan; 2 Geriatrics Research Education and Clinical Center, Puget Sound Health Care System, Seattle WA and Division of Gerontology and Geriatric Medicine, Department of Medicine, University of Washington, Bldg 1/Rm 810A, VAPSHCS, 1660 S. Columbian Way, Seattle, WA 98108, USA

**Keywords:** Blood–brain barrier, HIV-1, Pericytes, Brain endothelial cells, Cytokines, BMEC, AIDS, Neuroinflammation, Neuroimmune, Lipopolysaccharide

## Abstract

**Background:**

Human immunodeficiency virus-1 (HIV-1) enters the brain by crossing the blood–brain barrier (BBB) as both free virus and within infected immune cells. Previous work showed that activation of the innate immune system with lipopolysaccharide (LPS) enhances free virus transport both *in vivo* and across monolayer monocultures of brain microvascular endothelial cells (BMECs) *in vitro*.

**Methods:**

Here, we used monocultures and co-cultures of brain pericytes and brain endothelial cells to examine the crosstalk between these cell types in mediating the LPS-enhanced permeation of radioactively-labeled HIV-1 (I-HIV) across BMEC monolayers.

**Results:**

We found that brain pericytes when co-cultured with BMEC monolayers magnified the LPS-enhanced transport of I-HIV without altering transendothelial electrical resistance, indicating that pericytes affected the transcytotic component of HIV-1 permeation. As LPS crosses the BBB poorly if at all, and since pericytes are on the abluminal side of the BBB, we postulated that luminal LPS acts indirectly on pericytes through abluminal secretions from BMECs. Consistent with this, we found that the pattern of secretion of cytokines by pericytes directly exposed to LPS was different than when the pericytes were exposed to the abluminal fluid from LPS-treated BMEC monolayers.

**Conclusion:**

These results are evidence for a cellular crosstalk in which LPS acts at the luminal surface of the brain endothelial cell, inducing abluminal secretions that stimulate pericytes to release substances that enhance the permeability of the BMEC monolayer to HIV.

## Background

Human immunodeficiency virus-1 (HIV-1) enters the central nervous system (CNS) where it is relatively protected from anti-viral drugs [1-3]. Once in the CNS, HIV-1 has a number of neurotoxic effects that result in cognitive, hormonal, vascular, and homeostatic deficits [4-11]. HIV-1 in the CNS may also act as a source for re-infecting the periphery [12]. For these reasons, it is important to understand the mechanisms by which HIV-1 gains entry into the CNS.

HIV-1 must cross the blood–brain barrier (BBB) to enter the CNS. HIV-1 crosses the BBB both within infected immune cells by the Trojan horse mechanism [13,14] and also as free virus [15]. These mechanisms are not passive but are highly regulated by intracellular machinery. Crossing within infected immune cells involves a form of diapedesis and is STAT-1 and JNK-dependent [16,17]. In contrast, free virus entry is a multi-step transcytotic process [15,18,19] that is JNK-independent but does depend on p38 mitogen-activated protein kinase [20] and glycoprotein interactions [21] with the mannose-6-phosphate receptor [22]. Both of these mechanisms, diapedesis of infected immune cells and free virus transcytosis, are enhanced by inflammation. This is relevant as HIV-1 and its viral proteins gp120 and TAT can activate the immune system and the cells that form the BBB [23-25]. It is important therefore, to further study the mechanisms by which neuroinflammation can enhance viral permeability across the BBB.

Lipopolysaccharide (LPS) application offers a useful model for studying the effects of neuroimmune activation on HIV-1 transport across the BBB. LPS is derived from the bacterial cell wall of gram negative bacteria [26] and is found in the circulation of HIV-1 patients as a result of gastroenteropathy-related bacterial translocation [27]. LPS enhances both immune cell [14] and free virus [20] transport across the BBB. LPS induces cytokine release into the blood and an even more prolonged immune response within the CNS [28]. LPS has many effects on the BBB and its cellular components [29], including altering paracellular and transcytotic permeability, altering brain-to-blood and blood-to-brain transporters [30-35], and inducing cytokine release [36,37], actions similar to those induced by HIV-1 and its proteins [38-43]. LPS should also prove useful for studying the cells that influence and modulate the BBB that together with brain endothelial cells form the neurovascular unit.

The neurovascular unit consists of endothelial cells, astrocytes, microglia, and other cells which are in constant crosstalk to inform one another of CNS events and to regulate BBB function [44]. Cytokines and other immunoactive substances are important messengers in this crosstalk. The least studied of these cells is the pericyte [45] that is also the cell in closest proximity to brain endothelial cells. The pericyte is a pluripotent cell and lies under the basement membrane surrounding CNS capillaries [46-48] and is known to be in intimate crosstalk with other cells of the neurovascular unit such as the astrocyte [49]. The pericyte is in direct contact with brain endothelial cells through both peg and socket joints and gap junctions. Recently, the pericyte has been shown to take up the HIV-1 virus and to be a site of viral replication [50].

Here, we examined the influence of pericytes on free virus transport across monolayers of brain microvascular endothelial cells (BMEC) and crosstalk between BMEC and pericytes in two experimental paradigms. First, we determined whether co-culture of BMEC with pericytes would alter the ability of free HIV-1 to cross the BMEC monolayer under both basal and LPS-treated conditions. Second, we examined the ability of pericytes to secrete cytokines either constitutively or induced by abluminal secretions from BMECs exposed to LPS. Together, the results show that LPS stimulates a crosstalk between brain endothelial cells and pericytes that enhance permeation of HIV-1 across BMEC monolayers.

## Methods

### Radioactive labeling

HIV-1 (MN) CL4/CEMX174 (T1) prepared and rendered non-infective by aldrithiol-2 treatment as previously described [51,52] was a kind gift of the National Cancer Institute, NIH. The virus was radioactively labeled by the chloramine-T method, a method which preserves vial coat glycoprotein activity [53,54]. Two mCi of ^131^I-Na (Perkin Elmer, Boston, MA), 10 μg of chloramine-T (Sigma) and 5.0 μg of the virus were incubated together for 60s. The radioactively-labeled virus was purified on a column of Sephadex G-10 (Sigma, St. Louis, MO). Human serum albumin (5 μg) was labeled by the chloramine-T method with 1 mCi of ^125^I-Na (Perkin Elmer) and purified on a G-10 column.

### Primary cultures of BMECs

Primary cultures of MBECS and mouse brain pericytes were prepared from 8-week-old CD-1 mice. All experiments were approved by the local (VA St Louis) animal use committee. BMECs were isolated by a modified method of Szabó *et al*. [55] and Nakagawa *et al*. [56]. In brief, the cerebral cortices from 8-week-old CD-1 mice were cleaned of meninges and minced. The homogenate was digested with collagenase type II (200 U/mL; Invitrogen, Carlsbad, CA) and DNase I (30 U/mL; Sigma,) in Dulbecco’s modified Eagle’s medium (DMEM) (Invitrogen) containing 100 units/mL penicillin, 100 μg/mL streptomycin, 50 μg/mL gentamicin and 2 mM GlutaMAX™-I (Invitrogen) at 37°C for 40 min. The pellet was separated by centrifugation in 20% bovine serum albumin (BSA)-DMEM (1,000 × g for 20 min) to remove neuron and glial cells. The microvessels obtained in the pellet were further digested with collagenase/dispase (1 mg/mL; Roche, Mannheim, Germany) and DNase I (30 U/mL) in DMEM at 37°C for 30 min. Microvessel fragments containing pericytes and endothelial cells were separated by a 33% continuous Percoll (Amersham Biosciences, Piscataway, NJ) gradient centrifugation (1,000 × g for 10 min) collected and washed in DMEM (1,000 × g for 10 min). To obtain BMECs, the obtained microvessel fragments were seeded on 60 mm culture dishes (BD FALCON™, BD Biosciences, Franklin Lakes, NJ) coated with 0.05 mg/mL fibronectin (Sigma), 0.05 mg/mL collagen I (Sigma) and 0.1 mg/mL collagen IV (Sigma). They were incubated in DMEM/Nutrient mixture F-12 HAM (DMEM/F-12) (Invitrogen) supplemented with 20% plasma derived bovine serum (PDS, Animal Technologies, Tyler, TX), 100 units/mL penicillin, 100 μg/mL streptomycin, 50 μg/mL gentamicin, 2 mM GlutaMAX™-I and 1 ng/mL basic fibroblast growth factor (bFGF; Sigma) at 37°C with a humidified atmosphere of 5% CO_2_/95% air. The next day, the BMECs migrated from the isolated capillaries and started to form a continuous monolayer. To eliminate contaminating cells (mainly pericytes), BMECs were treated with 4 μg/mL puromycin (Sigma) for the first 2 days [57]. After 2 days of the treatment, puromycin was removed from the culture medium. Culture medium was changed every other day. After 7 days in culture, BMECs typically reached 80-90% confluence.

### Primary cultures of pericytes

Mouse brain pericytes were obtained by a prolonged, 2-week culture of isolated brain microvessel fragments under selective culture conditions because brain microvessel fragments contain pericytes [56,58,59]. Brain microvessel fragments were isolated as described above for microvessels, and then the obtained fragments were seeded on uncoated 60 mm culture dishes (BD FALCON™) in DMEM containing 20% fetal bovine serum (FBS), 100 units/mL penicillin, 100 μg/mL streptomycin, 50 μg/mL gentamicin, 2 mM GlutaMAX™-I and incubated at 37°C with a humidified atmosphere of 5% CO_2_/95% air. The culture medium was changed twice a week. After 14 days in culture, pericytes typically reached 80-90% confluence. Brain pericytes were characterized by positive immunostaining for a-smooth muscle actin and negative for von Willebrand factor and were used at passage 2–3.

### Monoculture and co-culture methods

**Figure 1:**
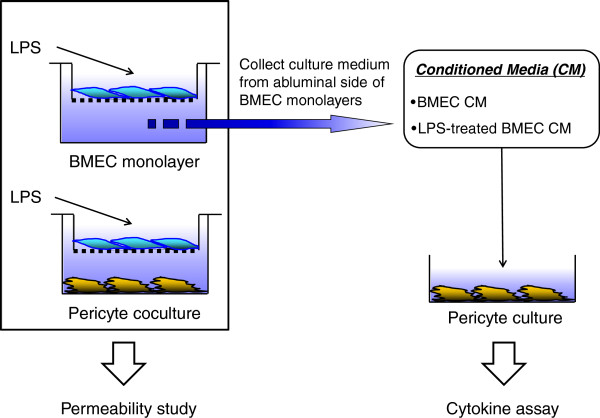
**Schematic diagram of *in vitro *BBB models and experimental design.** BMECs were seeded on the filter of Costar Transwell® inserts (BMEC monolayer). In pericyte co-culture, brain pericytes were cultured with BMECs using Transwell® insert without direct contact. Following treatment with LPS (10, 50, and 100 μg/mL) for 4 hr, both *in vitro* BBB models were used for HIV-1 transport studies. Conditioned media from the abluminal chamber of BMEC monolayer was collected for cytokine assay.

Monocultures of BMEC were used to produce conditioned media and to study I-HIV permeability. Monocultures of pericytes were used in cytokine assays, and co-cultures of BMECs with pericytes were used in I-HIV permeability studies (Table 1, Figure 1). BMEC monolayers were obtained by seeding BMECs (4 × 10^4^ cells/well) on the inside of the fibronectin-collagen IV (0.1 and 0.5 mg/mL, respectively)-coated polyester membrane (0.33 cm^2^, 0.4 μm pore size) of a Transwell®-Clear insert (Costar) and cultured in DMEM/F-12 supplemented with 20% PDS, 100 units/mL penicillin, 100 μg/mL streptomycin, 50 μg/mL gentamicin, 2 mM GlutaMAX™-I, 1 ng/mL bFGF and 500 nM hydrocortisone (Sigma) at 37°C with a humidified atmosphere of 5% CO_2_/95% air until the BMECs reached confluence (3 days). For monocultures of brain pericytes, cells were seeded (4 × 10^4^ cells/well) into 24-well culture plates (Costar, Corning, NY) and used after a few days.

**Table 1:** Effects of pericytes on transport rates of HIV-1(I-HIV) and albumin (I-Alb) and on transendothelial electrical resistance (TEER) in Monolayers of mouse brain microvascular Endothelial Cells (BMEC)

	**I-HIV**	**I-Alb**	**TEER**
BMEC: no pericytes	11.5 +/− 0.9 (15)	6.2 +/− 0.4 (15)	60 +/− 3 (15)
BMEC: + pericytes	9.0 +/− 0.4 (14)*	6.3 +/− 0.3 (14)	75 +/− 4 (15)*

Means are reported with their means =/- SE (n). I-HIV and I-Alb transport rates are in cm/min(10^-6^) and TEER is in Ω x cm^2^. *Statistically different from corresponding “no pericyte” group, *p*<0.05.

Co-culture of BMECs and brain pericytes was as described previously [58]; after a few days of pericyte culture as outlined above, BMECs (4 × 10^4^ cells/well) were seeded on the inside of the fibronectin-collagen IV (0.1 and 0.5 mg/mL, respectively)-coated polyester membrane (0.33 cm^2^, 0.4 mm pore size) of a Transwell®-Clear insert (Costar) placed in the well of a 24-well culture plate containing layers of brain pericytes (pericyte co-culture) and cultured for 3 days. To check the barrier integrity of BMECs in the BMEC monolayer and pericyte co-culture systems, transendothelial electrical resistance (TEER in Ω × cm^2^) was measured before the experiments and after an exposure of LPS using an EVOM voltohmmeter equipped with STX-2 electrode (World Precision Instruments, Sarasota, FL). The TEER of cell-free Transwell®-Clear inserts were subtracted from the obtained values.

### Measurement of permeability across BMEC

Lipopolysaccharide from *Salmonella typhimurium* (LPS; Sigma) was dissolved in serum-free DMEM/F-12 (DMEM/F-12 containing 1 ng/ml bFGF and 500 nM hydrocortisone) to concentrations of 10, 50, and 100 μg/ml. To initiate exposure of *in vitro* BBB models prepared as above to LPS, the culture medium containing serum in both the luminal and abluminal chambers was removed and then serum-free DMEM/F-12 was added to the abluminal chamber. Immediately, medium containing various concentrations of LPS (10, 50, 100 μg/ml) was added to the luminal chamber of the co-culture. Serum-free DMEM/F-12 without LPS was used as the control medium. TEER was measured at the end of 4 h incubation at 37°C. To initiate transport experiments, the medium was removed and BMECs were washed with physiological buffer containing 1% BSA (141 mM NaCl, 4.0 mM KCl, 2.8 mM CaCl_2_, 1.0 mM MgSO_4_, 1.0 mM NaH_2_PO_4_, 10 mM HEPES, 10 mM D-glucose and 1% BSA, pH 7.4). The physiological buffer containing 1% BSA was added to the outside (abluminal chamber; 0.6 mL) of the Transwell® insert and ^131^I-HIV-1 (3 × 10^6^ cpm/mL) was loaded into the luminal chamber. Samples were removed from the abluminal chamber at 15, 30, 60 and 90 min and immediately replaced with an equal volume of fresh 1% BSA/physiological buffer. The sampling volume from the abluminal chamber was 0.5 mL. All samples were mixed with 30% trichloroacetic acid (TCA; final concentration 15%) and centrifuged at 5,400 × g for 15 min at 4°C. Radioactivity in the TCA precipitate was determined in a gamma counter. The permeability coefficient and clearance of TCA-precipitable ^131^I-HIV-1 was calculated according to the method described by Dehouck *et al*. [60]. Clearance was expressed as microliters (μL) of radioactive tracer diffusing from the luminal to abluminal (influx) chamber and was calculated from the initial level of radioactivity in the loading chamber and final level of radioactivity in the collecting chamber:

$$\text{Clearance}\;\left(\mathrm{\mu L}\right)={\left[C\right]}_{C}\;\times \;V_{C}/{\left[C\right]}_{L},$$

where [C]_L_ is the initial radioactivity in a μL of loading chamber (in cpm/μL), C_C_ is the radioactivity in a μL of collecting chamber (in cpm/μL), and V_C_ is the volume of collecting chamber (in μL). During a 90-min period of the experiment, the clearance volume increased linearly with time. The volume cleared was plotted versus time, and the slope was estimated by linear regression analysis. The slope of clearance curves for the BMEC monolayer plus Transwell® membrane was denoted by PS_app_, where PS is the permeability × surface area product (in μL/min). The slope of the clearance curve with a Transwell® membrane without BMECs was denoted by PS_membrane_. The real PS value for the BMEC monolayer (PS_e_) was calculated from 1 / PS_app_ = 1 / PS_membrane_ + 1 / PS_e_. The PS_e_ values were divided by the surface area of the Transwell® inserts (0.33 cm^2^) to generate the endothelial permeability coefficient (P_e_, in cm/min).

### Cytokine detection

To obtain BMEC-conditioned media, the abluminal chamber of the BMEC monolayer was filled with 600 μL of serum-free DMEM/F-12. At the same time 100 μL of serum-free DMEM/F-12 (DMEM/F-12 containing 1 ng/ml bFGF and 500 nM hydrocortisone) without or with 100 μg/ml LPS was added to the luminal chamber of the BMEC monolayer as described above. After 4 h of exposure, the fluid in the abluminal chamber of the BMEC monolayer (600 μL) was collected as the BMEC conditioned media, sterilized by passing it through a 0.45 μm filter syringe, and stored at −80°C until use. Brain pericytes (4 × 10^4^ cells/well) were seeded on the wells of 24-well culture plate (Costar). We divided the pericyte cultures into 4 groups: pericytes treated with (1) BMEC conditioned medium, (2) LPS-treated BMEC-conditioned medium, (3) serum-free DMEM/F-12 containing no LPS (control), and (4) serum-free DMEM/F-12 containing LPS (100 μg/mL) where these media are from above. Pericytes were washed with serum-free DMEM/F-12, and then exposed to 200 μL of BMEC-conditioned medium, LPS-treated BMEC conditioned medium, or serum-free DMEM/F-12 with or without LPS (100 μg/mL) for 4 hr at 37°C. Culture supernatant (200 μL) was collected and stored at −80°C until use. The 22 cytokines [granulocyte colony-stimulating factor (G-CSF), granulocyte-macrophage colony-stimulating factor (GM-CSF), interferon gamma (IFN-g), interleukin-1 alpha (IL-1a), IL-1b, IL-2, IL-4, IL-5, IL-6, IL-7, IL-10, IL-12(p70), IL-13, IL-15, IL-17, interferon-inducible protein-10 (IP-10), keratinocyte chemoattractant (KC), monocyte chemoattractant protein-1 (MCP-1), macrophage inflammatory protein-1 alpha (MIP-1a), regulated upon activation, normal T-cell expressed and secreted (RANTES), and tumor necrosis factor-alpha (TNF-a)] in the collected supernatants, BMEC conditioned medium, and LPS-treated BMEC conditioned medium were measured with the mouse cytokine/chemokine Lincoplex® kit (Linco Research, St. Charles, MO) by following the manufacturer’s instructions.

### Statistical analysis

Values are expressed as means ± SEM. One-way and two-way analysis of variances (ANOVAs) followed by Tukey-Kramer’s test were applied to multiple comparisons in the permeability study. In the cytokine assay, one-way ANOVA followed by Tukey-Kramer’s test were applied to multiple comparisons among the control, BMEC conditioned medium, and LPS-treated BMEC conditioned medium groups. Student’s t-test was applied to comparison between the control and LPS groups. The differences between means were considered to be significant when *P* values were less than 0.05 using Prism 5.0 (GraphPad, San Diego, CA).

## Results

### Pericytes affect permeability

Table 1 shows that co-culturing with pericytes improved brain endothelial cell monolayer TEER but had no effect on albumin permeability. I-HIV crossed the BMEC monolayers with or without pericyte co-cultures faster than I-Alb despite the much larger size of I-HIV. Pericytes reduced the permeation of I-HIV across the BBB to a statistically significant degree (t = 2.60, df = 27, p<0.05). These results show that monolayers with or without pericytes are more permeable to HIV-1 than the much smaller albumin, demonstrating that HIV-1 is crossing BMEC by a process other than leakage. The results also show that pericytes have differing effects on the three mechanisms of permeability illustrated in this table: increasing TEER (decreasing paracellular permeability) and decreasing HIV permeability (adsorptive transcytosis), but not affecting albumin permeability (macropinocytosis).

### LPS affects permeability

**Figure 2:**
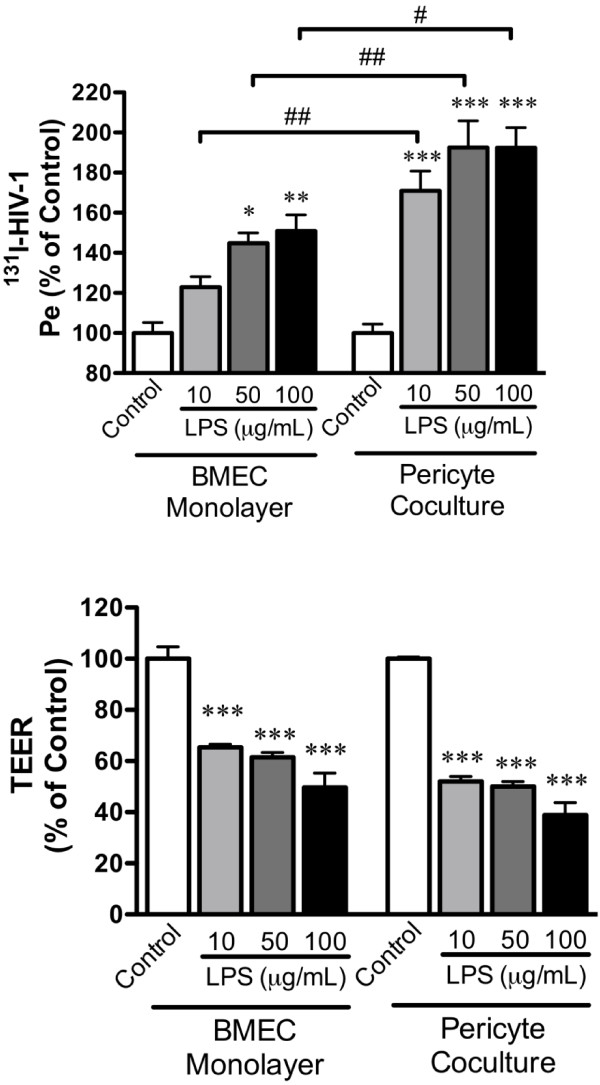
Effects of Pericytes on LPS-induced alterations in I-HIV permeability (Pe) and transendothelial electrical resistance (TEER). Upper panel: LPS increased I-HIV transport across the monolayers in comparison to respective controls (*p<0.05; **p<0.01; ***p<0.001). Pericytes enhanced the effect of any given dose of LPS on HIV-1 transport (#*p*<0.05; ##*p*<0.001). Lower panel: LPS decreased TEER (***p<0.001) with pericytes producing no modification of that effect.

Addition of LPS produced a dose-dependent increase in I-HIV-1 transport across the BMEC monolayer. Results (Figure 2) were expressed as percent of control in order to reduce statistical variance and to allow comparison across treatments and trials. For I-HIV, two-way ANOVA showed significant effects for culture conditions [with or without pericytes: F(1,76) = 29.5, *p*<0.001] and treatment [LPS concentration: F(3,76) = 27.5, *p*<0.001] with no effect for interaction. Tukey’s multiple comparison test showed that 50 and 100 μg/ml of LPS increased I-HIV transport across the monolayer in the absence of pericytes and that 10, 50, and 100 μg /ml of LPS increased I-HIV transport across the monolayer in the presence of pericyte co-culture. Additionally, any given concentration of LPS produced a statistically greater transport of HIV-1 in the monolayers co-cultured with pericytes when compared to the monocultures (Figure 2 upper panel).

LPS also decreased TEER (Figure 2, lower panel) as shown by the two-way ANOVA that found effects for culture conditions [F(1, 76) = 14.8, *p*<0.001] and LPS treatment [F(3,76) = 111.5, *p*<0.001]. However, Tukey’s multiple comparisons test found no effect of pericytes on the LPS-induced decrease in TEER.

### Effects of cytokine release

**Figure 3:**
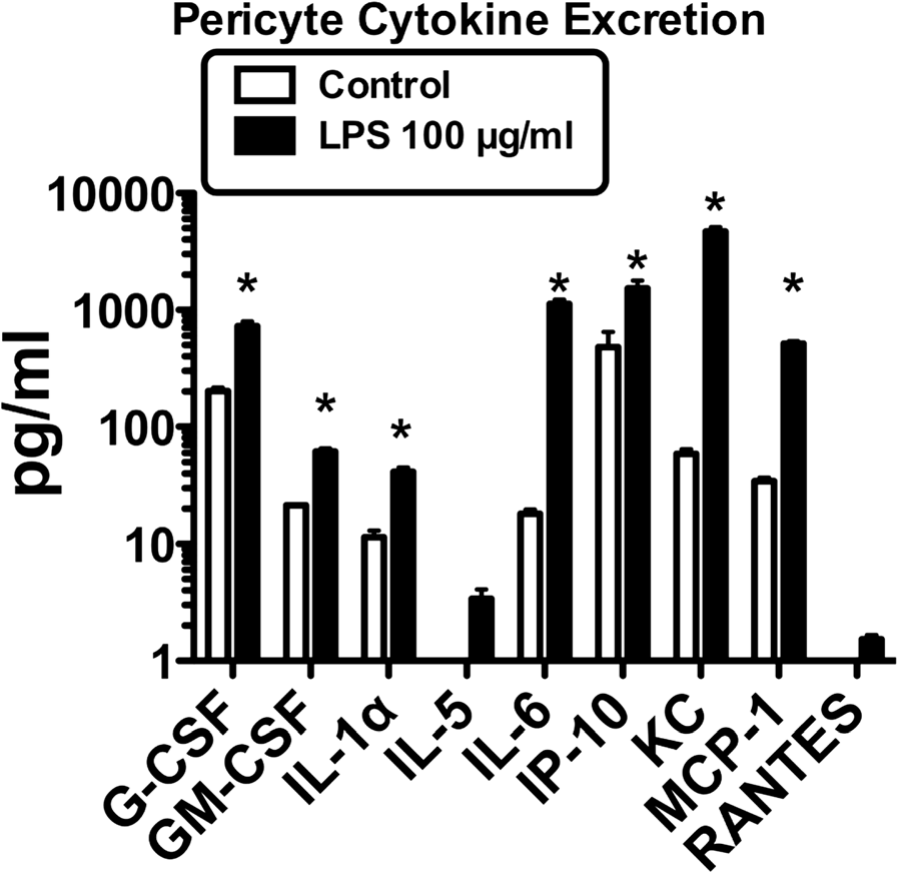
**Expression of cytokines by pericytes.** Constitutive expression of seven cytokines was found in the control group with increased expression of these and induction of two others after addition of LPS. GM-CSF: granulocyte-macrophage colony-stimulating factor, IL-1α: interleukin-1 alpha, IP-10: interferon-inducible protein-10, KC: keratinocyte chemoattractant, MCP-1: monocyte chemoattractant protein-1, RANTES: regulated upon activation, normal T-cell expressed and secreted.

To further investigate the mechanism by which pericytes, LPS, and brain endothelial cells interact, we determined whether culture media obtained from brain endothelial cells could induce cytokine release from pericytes. We determined the ability of pericytes to release cytokines either constitutively or when stimulated by LPS. Of 22 cytokines measured, seven (G-CSF, GM-CSF, IL-1 alpha, IL-6, IP-1-, KC, and MCP-1) were constitutively released by pericytes and two others (IL-5 and RANTES) were induced when pericytes were exposed to LPS for 4 h (Figure 3). LPS also caused a statistically significant increase in the release of each of the seven constitutively- released cytokines, *p*<0.01 (Figure 3).

### Endothelial-pericyte crosstalk

**Figure 4:**
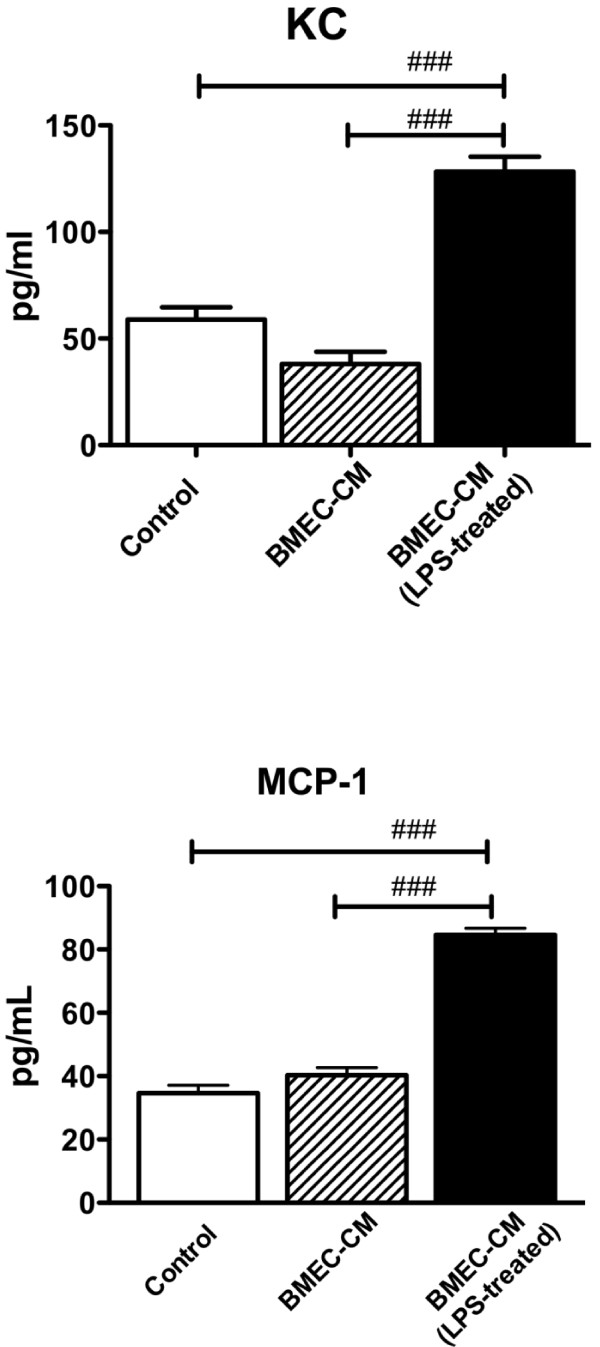
**Release of cytokines from pericytes.** Effects of culture media from brain endothelial cells (BMEC-CM) and culture media from brain endothelial cells exposed to LPS (BMEC- CM LPS-treated) on release of keratinocyte chemoattractant (KC) or monocyte chemoattractant protein-1 (MCP-1) from pericytes. ### *p*<0.001.

We then incubated pericytes with culture media obtained from brain endothelial cells; in some cases, the endothelial cells had been exposed to LPS. Table 2 shows that four cytokines were detectable in the endothelial cell-conditioned culture media. Of the nine cytokines released by pericytes (Figure 3), endothelial culture media only affected the release of two of them (Figure 4): KC [F(2,14) = 58.8, *p*<0.001] and MCP-1 [F(2,14) = 137, *p*<0.001]. In both cases, culture media from endothelial cells that had been exposed to LPS, but not culture media from cells that were unexposed, produced the increase.

**Table 2:** Concentrations of cytokines in abluminal culture media from BMECs exposed or not exposed to luminal LPS

	**G-CSF**	**IL-1 alpha**	**IP-10**	**KC**
No LPS	171 +/− 6	9.5 +/− 1.4	18.8 +/− 2.9	0
LPS	191 +/− 9	9.0 +/− 1.1	19.6 +/− 5.3	7.8 +/− 0.9

This culture media was subsequently added to cultures of pericytes (Figure 4).

Cytokines as designated in legend to Figure 3. Means +/− SE are shown, n = 5/cell. Units are pg/ml.

## Discussion

We first studied the effects of pericytes on the permeability of brain endothelial cell monolayers. The barrier function of brain endothelial cells occurs through a low level of transcytotic mechanisms (macropinocytosis and fenestrae), and an increase in paracellular mechanisms (intercellular tight junctions) [61]. To some degree, the mechanisms that control and alter transcytotic events are different from those controlling paracellular permeability [62,63]. Electrical resistance measured by TEER measures channel connections typically ascribed to paracellular permeation. Here, TEER was very low, consistent with little effect on paracellular channel formation. Albumin permeation, however, was also low, indicating that transcytotic mechanisms were likely to be low. Mature fenestrae would produce both a low TEER and high albumin permeation, so it is likely that fenestrae were also not present in large numbers in these cells. Co-culturing pericytes with the brain endothelial cells significantly improved TEER, suggesting improved tight junction function, but did not alter albumin permeation (Table 1). BMEC-pericyte co-cultures had significantly reduced permeation of I-HIV. Although the HIV-1 virus is much larger than the albumin molecule, the rate at which I-HIV crossed the brain endothelial cell monolayer was much faster than that of I-Alb regardless of whether the endothelial cells were co-cultured with pericytes. This is consistent with the vesicular process for HIV-1 transport being independent of nonspecific vesicular mechanisms such as macropinocytosis.

LPS produced an increase in I-HIV permeation in the monolayers co-cultured and not co-cultured with pericytes. LPS also decreased TEER (that is, it increased paracellular permeability); therefore, this increase in I-HIV could be mediated by transcytotic or paracellular mechanisms. However, the presence of pericytes magnified the LPS effect on HIV-1 permeability without affecting TEER. This suggests that the pericyte-dependent portion of LPS-enhanced I-HIV permeation is transcytotic rather than paracellular.

The above results show that pericytes facilitate the LPS-enhanced transport of HIV-1 across brain endothelial cells. We then investigated some of the mechanisms by which pericytes could mediate such facilitation. LPS crosses the *in vivo* BBB, even the disrupted BBB, poorly if at all [64]. *In vitro*, LPS produces different patterns of cytokine release when added to the luminal versus the abluminal chamber, consistent with an inability to cross the *in vitro* BBB as well [37,65]. We, therefore, postulated that LPS was likely acting at the luminal surface of the brain endothelial cell, rather than at the pericytes in the abluminal chamber, as the first step in a neuroimmune-based modulation of the crosstalk between pericytes and brain endothelial cells. More specifically, we postulated that LPS acts at the luminal surface of the brain endothelial cell to induce release of soluble factors from its abluminal surface. These soluble factors would then act on pericytes, inducing them to release soluble factors that would modulate brain endothelial cell transcytosis of I-HIV. To test this hypothesis, we examined the release of cytokines from BMECs and pericytes. It is known that both endothelial cells and pericytes release cytokines, that cytokines are important in communication between the cells of the neurovascular unit, that cytokines enhance HIV-1 transcytosis, and that LPS can act on one side of the BBB to affect the release of cytokines from the other side [37,44,66,67].

We first exposed the luminal surface of BMEC monolayers to a short, 4 h exposure to LPS, then collected the abluminal culture media from these monolayers and abluminal culture media from BMEC monolayers not exposed to LPS was used as a control. We then assessed the ability of the culture media to release cytokines from pericytes. We found that culture media from LPS-exposed brain endothelial cells did indeed increase the release of two cytokines, KC and MCP-1, from pericytes. The level of these cytokines was much higher in the pericyte culture medias (40–140 pg/ml; see Figure 4) than in the brain endothelial cell culture medias (see Table 2), thus ruling out contamination as a source of these results. Likewise, it is unlikely that LPS from the endothelial cell culture media caused this stimulation as the LPS was added to the luminal chamber of the endothelial cells, whereas the media exposed to the pericytes was from the abluminal chamber. Furthermore, LPS contamination would have been expected to produce the cytokine profile observed in Figure 3 and not just increases in KC and MCP-1. Therefore, the most parsimonious explanation of our results fits our hypothetical model: LPS acting at the luminal surface of brain endothelial cells stimulates release of soluble factors from their abluminal surface which then modulates pericyte release of immune-active factors.

The levels of release of KC and MCP-1 are substantial. Given that the pericyte cultures contained about 50 μg of protein, we estimate MCP-1 and KC concentrations of 960 and 1440 pg/mg protein. This exceeds the levels produced in brain after *in vivo* administration of LPS [67].

Although we found the release of two cytokines from pericytes was specifically affected, it is likely that the release of many other immune active substances is similarly modulated. However, pericyte-secreted MCP-1 could be directly involved in the enhanced transcytosis of HIV-1. MCP-1 derived from astrocytes [68] and microglia [69] mediates cross talk with brain endothelial cells that increases the diapedesis across the BBB of HIV-1 infected macrophages and monocytes. MCP-1 also affects voltage-gated potassium channels in brain endothelial cells [70]. Here, the evidence shows that pericytes are also a source of MCP-1 which is released in response to signals from brain endothelial cells. It could be that MCP-1 released from pericytes alters brain endothelial transcytotic processes that affect the permeation of free HIV-1.

## Conclusion

Pericytes potentiate the LPS-enhanced transport of HIV-1 across BMECs. This pericyte potentiation does not affect TEER and so is likely mediated through transcytotic mechanisms. We conclude that LPS acts at the luminal surface of the BMEC monolayer, inducing abluminal secretion of cytokines and other substances that in turn act on pericytes, the pericytes then secrete factors that enhance I-HIV transcytosis across the BMECs.

## Competing interests

The authors do not have financial or nonfinancial competing interests.

## Authors’ contributions

Both WAB and SD contributed to experimental design, statistical analysis, and writing of the MS. Experimental work was carried out by SD. All authors read and approved the final manuscript.
